# Forecasting PM_2.5_-induced lung cancer mortality and morbidity at county level in China using satellite-derived PM_2.5_ data from 1998 to 2016: a modeling study

**DOI:** 10.1007/s11356-020-08843-9

**Published:** 2020-04-23

**Authors:** Wei-Bin Liao, Ke Ju, Qian Zhou, Ya-Min Gao, Jay Pan

**Affiliations:** 1 West China School of Public Health and West China Fourth Hospital, Sichuan University, No. 17, Section 3, Ren Min Nan Road, Chengdu, 610041 Sichuan China; 2 Medical College, Northwest Minzu University, Lanzhou, China; 3 West China Research Center for Rural Health Development, Sichuan University, Chengdu, China

**Keywords:** Lung cancer, PM_2.5_, Mortality, Morbidity, China, Spatial analysis

## Abstract

The serious ambient fine particulate matter (PM_2.5_) is one of the key risk factors for lung cancer. However, existing studies on the health effects of PM_2.5_ in China were less considered the regional transport of PM_2.5_ concentration. In this study, we aim to explore the association between lung cancer and PM_2.5_ and then forecast the PM2.5-induced lung cancer morbidity and mortality in China. Ridge regression (RR), partial least squares regression (PLSR), model tree-based (MT) regression, regression tree (RT) approach, and the combined forecasting model (CFM) were alternative forecasting models. The result of the Pearson correlation analysis showed that both local and regional scale PM_2.5_ concentration had a significant association with lung cancer mortality and morbidity and compared with the local lag and regional lag exposure to ambient PM_2.5_; the regional lag effect (0.172~0.235 for mortality; 0.146~0.249 for morbidity) was not stronger than the local lag PM_2.5_ exposure (0.249~0.294 for mortality; 0.215~0.301 for morbidity). The overall forecasting lung cancer morbidity and mortality were 47.63, 47.86, 39.38, and 39.76 per 100,000 population. The spatial distributions of lung cancer morbidity and mortality share a similar spatial pattern in 2015 and 2016, with high lung cancer morbidity and mortality areas mainly located in the central to east coast areas in China. The stakeholders would like to implement a cross-regional PM_2.5_ control strategy for the areas characterized as a high risk of lung cancer.

**Electronic supplementary material:**

The online version of this article (10.1007/s11356-020-08843-9) contains supplementary material, which is available to authorized users.

## Introduction

Lung cancer is one of the most common causes of cancer morbidity and mortality worldwide; it accounts for about 11.6% of the total diagnosed cases and 18.4% of the total cancer deaths, with the age-standardized rate of 22.5 new incidence cases per 100,000 and age-standardized rate of 18.6 new death cases per 100,000, based on the GLOBOCAN estimates of global cancer incidence, mortality, and prevalence (Ferlay et al. 2019). China has one of the highest disease burden levels of lung cancer in the world (Torre et al. 2015). According to the latest Chinese cancer registration annual report, the raw incidence rate of lung cancer in 2014 was 77.42 per 100,000 men and 40.10 per 100,000 women.

A growing body of epidemiological studies showed that particulate matter (PM), especially fine particulate matter of aerodynamic diameter < 2.5μm (PM_2.5_), has an adverse effect on human health (Fajersztajn et al. 2013; Kampa and Castanas 2008), especially for lung cancer (Wang et al. 2019; Dehghani et al. 2017) as well as cardiovascular diseases (Zhang et al. 2014a, b; Beelen et al. 2014). What’s more, the International Agency for Research on Cancer (IARC) concluded that exposure to PM from outdoor air pollution is carcinogenic to humans (IARC group 1) and causes lung cancer (Loomis et al. 2013). Similar findings were also obtained in cohort studies from Europe, North America, and Asia. For example, in Europe, evidence from 17 cohorts suggested that a statistically significant association between risk for lung cancer incidence and PM_2.5_, the hazard ratio (HR), was 1.18 (0.96–1.46) per 5 μg/m^3^ (Raaschou-Nielsen et al. 2013). In the USA, a large cohort study examined associations between long-term ambient PM_2.5_ concentrations and lung cancer mortality in lifelong never smoke and found a 15–27% increase in lung cancer mortality for each > 10 μg/m^3^ increase in PM_2.5_ concentrations (Turner et al. 2011). In China, a 12-year cohort study conducted in northern China indicated that each 10 μg/m^3^ increase in PM_10_ concentration was associated with a 3.4–6.0% increase in lung cancer mortality, and the association was various in men and women (Li et al. 2018).

In addition to epidemiological study of lung cancer and PM_2.5_, the spatial distribution of PM_2.5_ also makes a specific contribution to lung cancer mortality and morbidity. As a consequence of reform and opening up, China has been experiencing high concentrations of air pollution (Brauer et al. 2012). The serious PM_2.5_ pollution issue in China has attracted great attention in recent years, and the large quantities of air pollutants indicate that PM_2.5_ pollution has expanded over a large regional scale (Li et al. 2017; Hu et al. 2014). Recent studies in some mega-cities of China have focused on the characteristics, chemical compositions, sources, and formation mechanism of PM_2.5_ (Li et al. 2019; Gao et al. 2018; Zheng et al. 2015). Specifically, densely populated mega-cities worsen the situation that the transport of pollutants may cross over geographical broader and contributes significantly to the formation of secondary aerosol (Huang et al. 2014). Moreover, due to a large amount of anthropogenic emissions, the impact of long-range transport of pollutants from China may affect the Pacific Ocean, other countries in Asia, and even North America (Wuebbles, Lei, and Lin 2007). Thus, these findings suggest that the regional transport of air pollutants may play an important role in the formation of PM_2.5_ pollution and spatial inequity of lung cancer.

To provide a reference to investigate the association between lung cancer mortality, morbidity, and long-term PM_2.5_ pollution, it is necessary to obtain detailed information about the local and regional variation of lung cancer mortality and morbidity and PM_2.5_ concentration. Therefore, in this study, we first investigated the association between lung cancer mortality and morbidity with local and regional PM_2.5_ concentrations. Secondly, the concentration-response functions between the annual lung cancer outcomes and long-term exposure to PM_2.5_ concentrations by five forecasting models were established. Finally, we obtained the forecasting spatial distribution of lung cancer mortality and morbidity in China.

## Materials and methods

### Materials and data processing

#### Lung cancer mortality and morbidity data

Lung cancer mortality and morbidity (ICD-10, C33-C34) were collected from the Chinese cancer registry annual report from 2009 to 2017, released by the National Central Cancer Registry (NCCR) and Disease Prevention and Control Bureau, Ministry of Health. The NCCR evaluated the registry data based on the criteria of quality control in the program protocol. According to the results of quality control, only data with good completeness and validity, according to the registries, were selected and analyzed for mortality and morbidity of cancer in China. All data on lung cancer mortality and morbidity are reported to population-based cancer registries in the centers for disease control, cancer hospital, or institute of cancer prevention and control.

A total of 1294 cancer registries were reported from 2006 to 2014; among those cancer registries, only few of the cancer registries published continuous data from 2006 to 2014; many new cancer registries reported cancer data during 2010–2014; thus, the whole lung cancer dataset was an unbalanced panel (see Table 1). By 2014, 339 cancer registries submitted data and qualified, with data distributed in 31 provinces and municipalities, including 129 urban areas and 210 rural areas. The population covered by cancer registration areas in 2014 was 288,243,347 (146,203,891 males and 142,039,456 females), which accounted for 21.07% of the entire national population. In the present study, cancer registries were not selected wherein the lung cancer mortality or morbidity was 0. Similarly, cancer registries were selected wherein lung cancer mortality or morbidity was great than 0. Besides, due to the small spatial scale of cancer registry areas, we grouped these areas into a large scale and recalculated the mortality and morbidity using their total combined population. Finally, a total of 1194 cancer registries from 2006 to 2014 were obtained in the present study, and the annual lung cancer mortality and morbidity were agreed, as the death and incidence of newly increasing lung cancer cases occurred in a single year for the covered population.

**Table 1:** Descriptions for cancer registration areas from 2006 to 2014

Year	No. of registries	No. of urban registries	No. of rural registries	Population (10 thousands)
2006	34	15	19	5956
2007	38	17	21	5980
2008	41	20	21	6613
2009	72	31	41	8447
2010	145	58	87	12,465
2011	177	77	100	14,575
2012	193	73	120	19,806
2013	255	88	167	22,649
2014	339	129	210	28,824

**Fig. 1:**
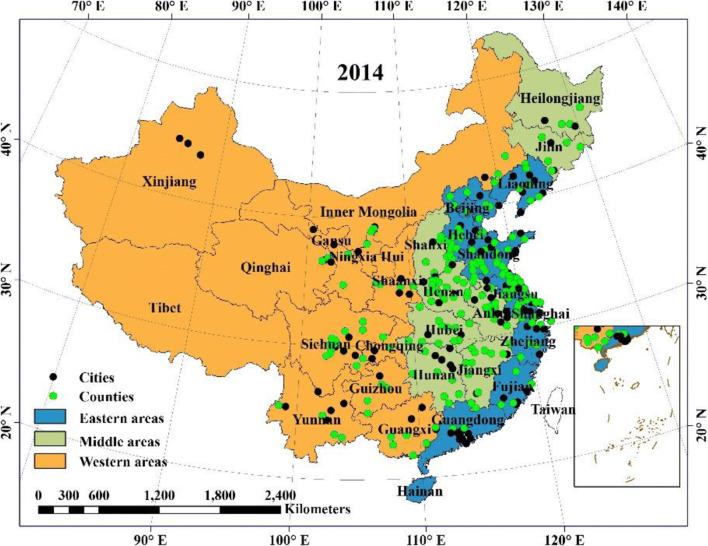
Spatial distribution of the cancer registries in 2014 across China

The classification of Eastern areas, Middle areas, and Western areas is based on the standard of the National Statistics Bureau. Eastern areas consist of Beijing, Tianjin, Hebei, Liaoning, Shanghai, Jiangsu, Zhejiang, Fujian, Shandong, Guangdong, and Hainan. Middle areas consist of Heilongjiang, Jilin, Shanxi, Anhui, Jiangxi, Henan, Hubei, and Hunan. Western areas consist of Inner Mongolia, Guangxi, Chongqing, Sichuan, Guizhou, Yunnan, Tibet, Shaanxi, Gansu, Qinghai, Ningxia, and Xinjiang. Figure 1 and Fig. S1 shows the spatial distribution of the cancer registries in this study.

#### Satellite-derived annual PM_2.5_ concentration data

The annual means of global PM_2.5_ concentration at 0.01° × 0.01° spatial resolution were collected from the Atmospheric Composition Analysis Group website of Dalhousie University. Van Donkelaar et al. estimated the global PM_2.5_ concentrations using multiple satellite products (MISR, MODIS Dark Target, MODIS and SeaWiFS Deep Blue, and MODIS MAIAC) (van Donkelaar et al. 2016; van Donkelaar et al. 2015).

To avoid the bias of uninhabited areas, such as the Taklimakan Desert, where few people live and the lung cancer mortality and morbidity are almost zero. Therefore, the dust- and sea salt-removed annual PM_2.5_ concentrations with 0.01° × 0.01° spatial resolution dataset were used in this study. For each cancer registry area, we first spatially matched the polygon shapefile of the cancer registry area with global surface annual PM_2.5_ concentration data and then calculated the annual mean PM_2.5_ concentration using the data of the grid point that fall within each cancer registry area from 1998 to 2016.

#### Conceptual framework

**Fig. 2:**
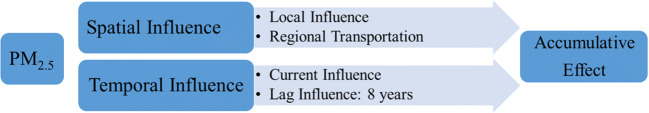
The conceptual framework

Based on the abovementioned background, we developed our conceptual framework and summarized it in Fig. 2. We proposed that the mortality and morbidity of lung cancer were not only under the influence of local PM_2.5_ pollution, but also the regional PM_2.5_ pollution. Figure 2 displays the conceptual framework of this study, with spatial influence representing the PM_2.5_ concentration from the local area and surrounding areas, temporal influence representing the lag effect in PM_2.5_ concentration, and lung cancer exposure-response relationship.

In this study, a contiguity-based conceptualization was constructed, where the definition of the neighborhood is based on sharing a common boundary or node for a specific area. Then, the mean value of all surrounding PM_2.5_ concentrations represented the regional scale. The definition of regional scale can be determined as follows:

1 $$ \mathrm{reg}=\frac{\sum_{i=1}^n\mathrm{P}{\mathrm{M}}_{2.{5}_i}}{n} $$

where PM_2.5_ is the area unit concentration, *n* is the total number of neighborhoods, and *i* is a certain neighbor. More importantly, previous studies have revealed the relationship between time lag from the exposure of PM_2.5_ and the development of lung cancer mortality and morbidity (Han et al. 2017; Sloan et al. 2012; Biggeri et al. 2005). It is confirmed that prolonged high exposure to PM_2.5_ adversely influences lung cancer risk. Moreover, studies from the major cities of China have shown that the time lag of PM_2.5_ exposure to lung cancer morbidity and/or mortality is 7 to 8 years (Chen, Li, and Zhou 2003; Zhang 2014a, b). Therefore, in this study, two group variables represent the regional effect and lag effect in PM_2.5_ concentration and lung cancer exposure-response relationship. The definition of exposure-response relationship can be determined as follows:

2 $$ {Y}_{t,i}=\alpha +\beta \left(\mathrm{loc}\right)+\lambda \left(\mathrm{reg}\right)+{\varepsilon}_{t,i} $$

where *Y*_*t*, *i*_ is the mortality or morbidity of lung cancer on year *t* at area *i*. *α* is the intercept, loc is the local PM_2.5_ concentration in the current year and previous 8 years (termed loc-lag0, lag1, lag2, lag3, lag4, lag5, lag6, lag7, and lag8, respectively), reg is the regional PM_2.5_ concentration in the current year and previous 8 years (termed reg-lag0, lag1, lag2, lag3, lag4, lag5, lag6, lag7, and lag8, respectively), and *ε*_*t*, *i*_ is the error term. The Pearson correlation analysis was used to evaluate the association between lung cancer outcomes (lung cancer mortality and morbidity) and annual PM_2.5_ concentrations of local lag and regional lag in the current year and the previous 8 years.

### Methods

#### Spatial autocorrelation analysis

The Globe Moran’s *I* is a measure of spatial autocorrelation developed by Moran (Moran 1950). The Globe Moran’s *I* has been widely used in public health to investigate spatial clusters of cancer (Zhang and Nitin 2018; Kulldorff et al. 2006). We figured out the Globe Moran’s *I* statistics in ArcGIS 10.1 to examine the spatial autocorrelation of lung cancer mortality and morbidity and the PM_2.5_ concentration in China. The equation for Moran’s *I* statistic is as follows:

3 $$ I=\frac{n}{S_0}\frac{\sum_{i=1}^n{\sum}_{j=1}^n{w}_{ij}\left({x}_i-\overline{x}\right)\left({x}_j-\overline{x}\right)}{\sum_{i=1}^n{\left({x}_i-\overline{x}\right)}^2} $$

where *n* is the number of spatial units; *x*_*i*_ or *x*_*j*_ is the mortality or morbidity of lung cancer; the PM_2.5_ concentration in area *i*, *j*, and *w*_*ij*_ is a matrix of spatial weight between area *i* and *j*; and *S*_0_ is the sum of all *w*_*ij*_:${S}_0={\sum}_{i=1}^n{\sum}_{j=1}^n{w}_{ij}$. The value of Moran’s *I* usually ranges from − 1 to 1. *Z*-statistics is used to test the significance of Moran’s *I*: *Z* = *I* − *E*[*I*]/*STD*[*I*], where *E*[*I*] =  − 1/(*n* − 1), STD = *E*[*I*^2^] − *E*[*I*]^2^.

The spatial relationships among counties or cities were characterized by the spatial weight matrix (Anselin 2013). In our study, an inverse distance matrix, which defines the impact of one feature on another feature and decreases with distance, was used for spatial weights. An inverse distance weights matrix was constructed in ArcGIS by using the county-level point-shape file. To our knowledge, there is no consistent evidence showing a fixed distance to characterize the impact of PM_2.5_, and the variation that distance may have an influence on Moran’s *I*. Thus, we prefer a default distance to construct the weight matrix.

#### Forecasting models

As aforementioned, due to the influence of regional transportation of PM_2.5_ pollutant, two group variables were used to establish the five alternative forecasting models, including the ridge regression model, the partial least squares regression model, the model tree-based regression model, the regression tree approach, and the combination forecasting model. Text S1 provided detailed information about five forecasting models. To evaluate the performance of alternative models, the error analysis was conducted to measure the accuracy between the observed and predicted lung cancer mortality and morbidity. Mean square error (MSE), mean absolute error (MAE), mean absolute percentage error (MAPE), Theil inequality coefficient (Theil IC), bias proportion (BP), variance proportion (VP), and covariance proportion (CP) are model evaluation indices which were used to measure the error of the lung cancer mortality and morbidity. These evaluation indices are expressed as

$$ \mathrm{MSE}=\frac{1}{n}{\sum}_{i=1}^n{\left({y}_i-\hat{y_i}\right)}^2,\kern0.5em \mathrm{MAE}=\frac{1}{n}{\sum}_{i=1}^n\left|{y}_i-\hat{y_i}\right|,\kern0.5em \mathrm{MAPE}=\frac{1}{n}{\sum}_{i=1}^n\left|\frac{y_i-\hat{y_i}}{y_i}\right|\cdotp 100\%,\kern0.5em \mathrm{Theil}\ \mathrm{IC}=\frac{\sqrt{\frac{1}{n}{\sum}_{i=1}^n{\left({y}_i-\hat{y_i}\right)}^2}}{\sqrt{\frac{1}{n}{\sum}_{i=1}^n{y_i}^2}+\sqrt{\frac{1}{n}{\sum}_{i=1}^n{{\hat{y}}_i}^2}},\mathrm{BP}=\frac{{\left(\overline{\hat{y_i}}-\overline{y_i}\right)}^2}{\sum_{i=1}^n{\left(\hat{y_i}-{y}_i\right)}^2/n},\kern0.75em \mathrm{VP}=\frac{{\left({\sigma}_{\hat{y_i}}-{\sigma}_{y_i}\right)}^2}{\sum_{i=1}^n{\left(\hat{y_i}-{y}_i\right)}^2/n},\kern0.5em \mathrm{and}\kern0.5em \mathrm{CP}=1- BP- CP $$

where *y*_*i*_ is the observed value, ${\hat{y}}_i$ is the predicted value, $\overline{y_i}$ is the mean value of *y*_*i*_, $\overline{{\hat{y}}_i}$ is the mean value of ${\hat{y}}_i$, and ${\sigma}_{y_i}$ and ${\sigma}_{\hat{y_i}}$ are the standard deviation of predicted and observed lung cancer mortality and morbidity. The smaller value of MSE, MAE, MAPE, Theil IC, BP, and CP indicates the lower error for predicted value and the larger value of CP means better consistency between the forecast and observed lung cancer mortality and morbidity.

To avoid selection bias, first, we randomly divided lung cancer data into a training set and a testing set (90% training and 10% testing), and 10% of the lung cancer data was used to verify the alternative forecasting models. Then, we conducted the aforementioned forecasting process for 1000 loops as cross-validation. Thus, the mean value and standard deviation of the result of error analysis were given in this study, besides various proportion of training and testing sets for further verification (85%, 80%, 75%, and 70% for the training set; 15%, 20%, 25%, and 30% for the testing set). Then, the best performing model was used to construct the forecasting model, based on lung cancer mortality and morbidity from 2006 to 2014. Finally, annual lung cancer mortality and morbidity for 2408 counties across China from 2015 to 2016 were obtained by the forecasting model, and the gridded spatial distributions of lung cancer mortality and morbidity in 2015 to 2016 were obtained by using the Kriging interpolation in ArcGIS 10.2.

## Results

### Lung cancer mortality and morbidity in China

There were a total of 720,563 and 595,002 lung cancer morbidity and mortality cases reported in China from 2006 and 2014, of which 481,413 (66.8%) were male and 239,277 (33.2%) were female morbidity, and 404,679 (68.01%) were male and 190,323 (31.99%) were female mortality. Within both male and female groups, it was shown that urban areas have higher rates of lung cancer morbidity and mortality. Table S1 shows the morbidity and mortality of lung cancer from 2006 to 2014. The crude rate of lung cancer morbidity and mortality showed an increasing trend in this period. Figure S2 presents the spatial distribution of morbidity and mortality of lung cancer in 2014 in 307 registries. Figures S3-S4 show the spatial distribution of morbidity and mortality of lung cancer in China from 2006 to 2013. There were obvious spatial variations in the rate of lung cancer morbidity and mortality. The highest rates of lung cancer morbidity and mortality mainly located in the provinces of Liaoning, Shandong, Hebei, and Jiangsu, which are located in the eastern area of China.

### Spatial distribution of PM_2.5_

**Fig. 3:**
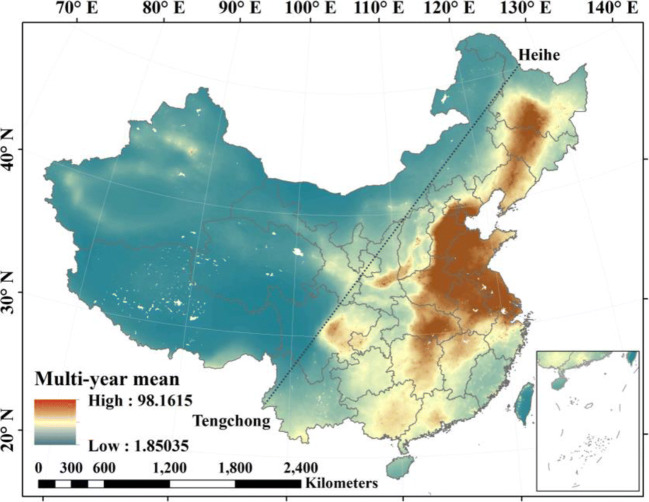
The spatial distribution of the multi-year (1998–2016) average PM_2.5_ concentration in China

The spatial distribution of the multi-year (1998–2016) average PM_2.5_ concentration in China is shown in Fig. 3. Most of the high PM_2.5_ concentration appeared in densely populated areas east of the Hei-Tengchong Line, which was proposed by Hu Huanyong in 1935 to illustrate China’s demographic distribution. There was a significant difference in the spatial patterns of the annual mean PM_2.5_ for eastern and western China. The overall average PM_2.5_ concentration for eastern China was two times higher than that for western China. For 1194 cancer registry areas, 1192 areas had annual mean PM_2.5_ concentrations greater than 10 μg/m^3^, the WHO air quality guidelines (AQG) recommendation for PM_2.5_ annual mean concentrations. There were 1167 cancer registry areas with annual mean PM_2.5_ concentrations greater than 15 μg/m^3^ (WHO Interim target-3 (IT-3)), 1033 cancer registry areas with annual mean PM_2.5_ concentrations greater than 25 μg/m^3^ (WHO Interim target-2 (IT-2)), and 859 cancer registry areas with annual mean PM_2.5_ concentrations greater than 35 μg/m^3^ (WHO Interim target-1 (IT-1)), that means more population were exposed to high levels of PM_2.5_ concentration.

### Pearson correlation analysis

The results from the Pearson correlation analysis showed a positive and significant association between lung cancer outcomes (mortality and morbidity) and the two group variables at the 0.01 level. As seen in Table 2, the correlation of the current year PM_2.5_ concentration was slightly lower than the lag correlations. Compared with the local lag and regional lag exposure to ambient PM_2.5_, the regional lag effect was not stronger than the local lag PM_2.5_ exposure, which may depend on the apportionment of surrounding PM_2.5_ concentration. Generally, both local and regional lag had long and positive effects on lung cancer mortality and morbidity.

**Table 2:** The Pearson correlation degree between lung cancer outcomes (mortality and morbidity) and PM2.5 concentration in China from 2006 to 2014

Lag	Lag 0	Lag 1	Lag 2	Lag 3	Lag 4	Lag 5	Lag 6	Lag 7	Lag 8
Mortality	0.249	0.251	0.247	0.272	0.286	0.291	0.299	0.287	0.294
Morbidity	0.215	0.225	0.217	0.244	0.265	0.280	0.299	0.289	0.301
Spatial lag	Slag 0	slag 1	slag 2	Slag 3	Slag 4	Slag 5	Slag 6	Slag 7	Slag 8
Mortality	0.172	0.185	0.187	0.217	0.220	0.224	0.233	0.221	0.235
Morbidity	0.146	0.164	0.161	0.194	0.204	0.221	0.238	0.231	0.249

Correlation is significant at the 0.01 level

### Globe spatial autocorrelation

The Globe Moran’s *I* statistics showed that the mortality and morbidity of lung cancer exhibited significant spatial autocorrelation for each year in China. The mortality and morbidity of lung cancer showed positive spatial autocorrelation of less than a 1% significance level, according to the results of Global Moran’s *I* statistics (as presented in Table S2). On the other hand, the annual PM_2.5_ concentration also had significant and positive spatial autocorrelation for each year (see Table S3). As shown, an increasing trend was found in the *Z*-score of Globe Moran’s *I* statistics, which indicates that it is less possible for PM_2.5_ in a region to follow the random distribution. Higher Moran’s *I* statistics of PM_2.5_ concentration, which were observed in 2009–2014, ranged from 0.525 to 0.708.

### Forecasting PM_2.5_-induced lung cancer mortality and morbidity

As shown in Table 3, the lung cancer mortality and morbidity prediction by the combination forecasting model had the lowest MSE, MAE, MAPE mean value, and higher CP value among the five forecasting models, which indicates that the combination forecasting model was performed better than the other models. The results of the sensitivity analysis (see Table S4 and S5), where various proportion for training and testing data sets was used to test the validation of five alternative forecasting models, further confirmed that the combination forecasting model was the best performed.

**Table 3:** Model evaluation of five alternative forecasting models

	RR	PLSR	RT	MT	CFM
Mortality					
MAE	11.08 (0.75)	11.16 (0.78)	11.38 (0.79)	11.08 (0.83)	10.89 (0.76)
MSE	195.96 (25.42)	199.28 (26.38)	207.67 (27.95)	201.32 (30.45)	190.54 (25.86)
MAPE	0.27 (0.02)	0.28 (0.02)	0.28 (0.02)	0.29 (0.04)	0.27 (0.02)
THEIL	1.11 (0.28)	1.03 (0.29)	0.68 (0.21)	0.78 (0.22)	1.08 (0.26)
BP	0.01 (0.01)	0.01 (0.01)	0.01 (0.01)	0.01 (0.02)	0.01 (0.01)
VP	0.45 (0.05)	0.53 (0.06)	0.30 (0.07)	0.31 (0.0982)	0.48 (0.07)
CP	0.45 (0.05)	0.46 (0.06)	0.69 (0.07)	0.68 (0.0986)	0.69 (0.07)
Morbidity					
MAE	12.78 (0.90)	12.79 (0.91)	13.09 (0.92)	13.12 (1.03)	12.5 (0.90)
MSE	268.26 (39.43)	268.86 (39.51)	282.71 (42.13)	288.41 (45.92)	260.34 (39.32)
MAPE	0.26 (0.02)	0.26 (0.02)	0.26 (0.02)	0.28 (0.04)	0.25 (0.02)
THEIL	1.12 (0.28)	1.02 (0.26)	0.71 (0.21)	0.67 (0.22)	1.09 (0.25)
BP	0.01 (0.01)	0.01 (0.01)	0.01 (0.01)	0.03 (0.04)	0.01 (0.02)
VP	0.45 (0.06)	0.50 (0.06)	0.3 (0.08)	0.28 (0.08)	0.48 (0.07)
CP	0.45 (0.06)	0.49 (0.06)	0.7 (0.08)	0.69 (0.09)	0.51 (0.07)

Values in parentheses are standard deviation

**Fig. 4:**
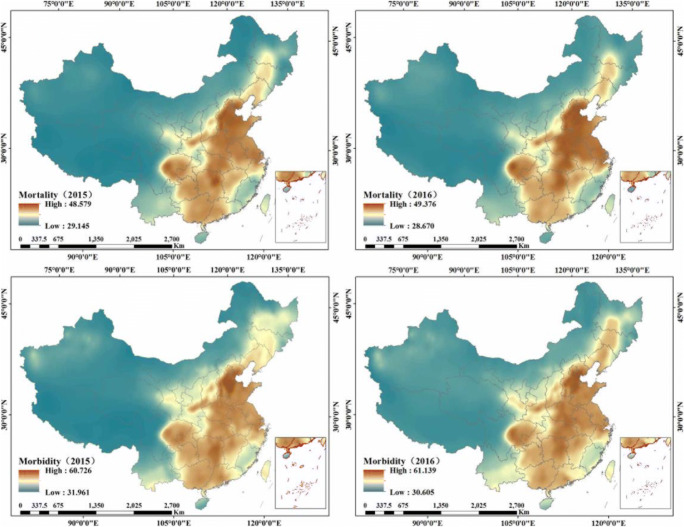
Forecasted lung cancer mortality and morbidity in China from 2015 to 2016

Figure 4 illustrates the combination forecasting model for lung cancer mortality and morbidity, due to exposure to PM_2.5_, where the red color represents high lung cancer mortality and morbidity and the blue color indicates low lung cancer mortality and morbidity. The overall forecasting lung cancer morbidity and mortality were 47.63, 47.86, 39.38, and 39.76 per 100,000 population. In general, the mortality and morbidity of lung cancer show distinct spatial patterns and strong variations across China. High lung cancer mortality and morbidity rates were observed in the north China plain, central China, the Sichuan Basin, and the Guangdong-Guangxi regions. In addition, the spatial distribution of lung cancer mortality and morbidity shows similar patterns for PM_2.5_ concentration.

## Discussions

In this study, we aim to forecast the spatial distribution of lung cancer morbidity and mortality in China in 2015 and 2016 due to exposure to PM_2.5_ concentration. We found that the current and previous 8-year PM_2.5_ concentration of local area and surrounding areas was significantly associated with lung cancer mortality and morbidity across China. This enables us to establish several statistical forecast models using both local and surrounding PM_2.5_ concentrations to predict the spatial distribution of lung cancer mortality and morbidity in China. Results showed that the combined forecasting model identified the best performance among five alternative models, besides a similar spatial distribution in morbidity and mortality of lung cancer in 2015 and 2016, with high lung cancer morbidity and mortality areas mainly located in the central to east coast districts.

In recent years, the occurrence of PM_2.5_ pollution has caught much attention and it is recognized that severe PM_2.5_ pollution is not a phenomenon specifically localized to a county or city (Li et al. 2014). Rather, previous studies have illustrated that PM_2.5_ pollution is the result of local pollutants superposed on background regional pollution, which has now expanded into a larger problem of a regional scale (Hu et al. 2014; Fu et al. 2008). Additionally, when meteorological conditions are favorable for the regional transportation of PM pollutants, this leads to a regional scale of PM pollution. In China, PM pollution is strongly affected by anthropogenic activities, including power generation, industrial processes, fossil and biomass fuel or agricultural waste combustion, and vehicle exhaust emissions (Li et al. 2014). Massive amounts of primary PM and high emissions of gas pollutants in densely distributed mega-cities have worsened PM pollution, which has contributed to regional air pollution (Zheng et al. 2015). Moreover, once regional pollution is formed, areas within the region cannot mitigate their pollution solely by reducing local emissions.

Given that the PM_2.5_ has been demonstrated to be a contributor to lung cancer, it can be concluded that the impact of PM_2.5_ regional transport on lung cancer should also be considered. In the present study, we found a significant association between lung cancer outcomes (mortality and morbidity) and PM_2.5_ from regional areas. Furthermore, compared with the local lag exposure to ambient PM_2.5_, the regional lag effect was not stronger than the local PM_2.5_ exposure. One of the main reasons we have taken the regional lag effect of PM_2.5_ pollutant into consideration was internal migration. According to the census in 2010 and national population sampling surveys of 2005 and 2015, nearly 66.64% of internal migrants traveling within provinces and most of them migrate to BTH, the Yangtze River delta, and the Pearl River Delta. Thus, the surrounding areas PM_2.5_ pollutant would like to exert influence on local lung cancer mortality and morbidity indirectly. Importantly, with the reform of the household register system (*hukou* system), there has been massive internal migration from rural to prosperous economic regions, as people seek higher income and better lifestyle opportunities in China (Mou et al. 2013). As a result, the regional transport of PM_2.5_ pollution appeared to be a factor leading to growing lung cancer mortality and morbidity.

In the present study, the short- and long-term exposure to PM_2.5_ concentration was used to establish five forecasting models. However, it is noteworthy that there would exist multi-collinearity problem when two or more predictor variables in a statistical model are linearly related. Furthermore, due to the changed patterns of collinearity, statistical inference using various geographic scales of sampled data leads to serious errors (Dormann et al. 2013). Based on the results of five forecasting models, the combined forecasting model identified the best performance in the lung cancer mortality and morbidity forecast. Consistent with the previous research, the combined forecasting model could improve forecast accuracy that the component forecasts contain useful and independent information (Armstrong 2001). Generally, the combined forecasts require deciding which forecasting model to include. Due to a lack of good knowledge on which forecasting method should be included and in order to generate independent forecasting combinations, we combined two types of forecasting methods: linear regression models and machine learning methods. Existing works on the multi-collinearity problem found that penalized methods such as ridge and lasso were performed well (Vigneau et al. 1997; Tibshirani 1996). Thus, we used the coefficient of the variation method rather than equal weight to establish the combined forecasting model.

One interesting founding is that the ridge regression and partial least squares regression were outperformed model tree and regression tree. When modeling the PM-lung cancer relationship, one important issue that needs to be considered is how to interpret the PM exposure accurately and precisely. However, those machine learning methods where the parameter estimation methods are quite different and do not provide interpretable parameter estimates and standard errors (Hastie 2008). More importantly, compared with linear regression methods, machine learning methods also have the same problems that the model is sensitive to slight changes in data set, due to multi-collinear among variables; it is difficult to interpret the parameter estimation and to separate the effects of multi-collinearity (Shan, Paull, and McKay 2006).

There are some limitations. Firstly, in the present study, the mean values of the PM_2.5_ concentration of neighborhoods were used to characterize the impact of regional transportation of PM_2.5_ over a short distance. However, it is noted that the PM_2.5_ were either from nearby cities, towns, and villages or from remote areas through long-range transport. Meanwhile, the regional transportation of PM_2.5_ was also influenced by wind directions. Therefore, under the different distances and wind directions, we could hardly quantify the impact of regional transportation of PM_2.5_ by the mean value. Secondly, to obtain a more realistic spatial distribution of lung cancer morbidity and mortality, the dust- and sea salt-removed annual PM_2.5_ concentration datasets were used to establish the forecasting models. Thus, we might underestimate the predicted lung cancer morbidity and mortality. Finally, we did not control the smoking population, as smoking data at the county or city level were not available.

## Conclusions

In summary, the present study demonstrated that the short- and long-term exposure to PM_2.5_ concentrations of the local area and regional areas were significantly associated with lung cancer mortality and morbidity across China, and compared with the local lag and regional lag exposure to ambient PM_2.5_, the regional lag effect (0.172~0.235 for mortality; 0.146~0.249 for morbidity) was not stronger than the local lag PM_2.5_ exposure (0.249~0.294 for mortality; 0.215~0.301 for morbidity). Results from spatial autocorrelation showed that the mortality and morbidity of lung cancer and PM_2.5_ concentration were significantly and spatially correlated for each year in China. Meanwhile, we also found a spatial association between lung cancer morbidity and mortality and satellite-derived PM_2.5_ concentration. Additionally, the sensitivity analysis and forecasting model in the present study provide a useful tool in the risk assessment of PM_2.5_. The spatial-temporal distribution of lung cancer morbidity and mortality and PM_2.5_ concentration will provide scientific support to government agencies and stakeholders. Furthermore, due to the impact of regional transportation on M_2.5_ and internal migration, it is necessary to strengthen regional collaborative pollution management among the high PM_2.5_ concentration regions.

## Electronic supplementary material

ESM 1 (DOCX 5002 kb)

## Data Availability

The data used in this study all come from published articles, yearbooks, and publicly accessible websites; therefore, all the data are open to everyone. No additional data is available.
