# Supplementary material for: Forecasting PM2.5-induced lung cancer mortality and morbidity at county level in China using satellite-derived PM2.5 data from 1998 to 2016: a modeling study
Source: Environ Sci Pollut Res Int. 2020 Apr 23;27(18):22946–55. doi: 10.1007/s11356-020-08843-9 (PMC7293676; doi:10.1007/s11356-020-08843-9)
Supplement: Supplementary file 1 — (DOCX 5002 kb) [file 11356_2020_8843_MOESM1_ESM.docx]

## Forecasting PM_2.5_-induced lung cancer mortality and morbidity at county-level in China using satellite-derived PM_2.5_ data from 1998 to 2016: a modelling study

Wei-Bin Liao^1^, Ke Ju^1^, Ya-Min Gao^2^ ,Qian Zhou^1^, Jay Pan^1,3╀^

^1^ West china school of public health, Sichuan University, Chengdu, China;

^2^ Medical College, Northwest Minzu University, Lanzhou, China;

^3^ West China Research Center for Rural Health Development, Sichuan University, Chengdu, China.

## ^╀^Corresponding to:

Prof. Jay Pan, West China School of Public Health, Sichuan University, No. 17, Section 3, Ren Min Nan Road, Chengdu, Sichuan 610041, China; Email: [panjie.jay@scu.edu.cn](mailto:panjie.jay@scu.edu.cn)

**S1. Forecasting models**

S1.1 Ridge regression

Ridge regression is a technique for estimating the regression coefficients with smaller mean square error than their least square counterparts, when variables are correlated(Hoerl and Kennard 1970). By adding small positive quantities to the regression estimates, ridge regression reduces the standard errors. That is,

$(\widehat{\beta_{0}}^{(ridge)},\widehat{\beta}^{(ridge)})=Min\sum_{i}^{n} \left[(y_{i}-\beta_{0}-\beta_{i}x_{i})^{2}+\lambda\sum_{j=1}^{p} \beta_{j}^{2}\right]$ (1)

$\widehat{\beta}(K)=(X^{T}X+kI)^{-1}X^{T}Y$ (2)

Where *k* is the ridge parameter less than one, noting that one of the main problems in using ridge regression is in choosing an appropriate value of *k*. Usually, the regression coefficients will vary widely for small values of *k* and then stabilize. It is necessary to choose the smallest value of *k* for which the regression coefficients seem to remain constant. A number of methods have been proposed to estimate *k*, based on data (Hoerl, Kennard et al. 1975, Lawless and Wang 1976, Cule and De Iorio 2012). In this study, we used a semi-automatic method to make a choice of ridge parameter, and the ridge regression model was performed with the *lmridge* package based on R software (Version 3.4.4).

S1.2 Partial least squares regression

The partial least squares regression (PLSR) is a statistical method that combines features and generalizes principal component analysis (PCA) and multiple linear regression. Its goal is to analyze or predict a set of dependent variables from a set of independent variables or predictors. This prediction is achieved by extracting from the predictors a set of orthogonal factors, called latent variables, which have the best predictive power. In the present study, the partial least squares regression model was established in R 3.4.4 with the package of *plr*, and a ‘leave-one-out’ cross-validation was performed.

S1.3 Regression tree

The regression tree is a machine-learning method for the construction and prediction of models from data (Breiman 2017). The prediction models are obtained by recursively partitioning the whole data and fitting a sample prediction model for each partition. The predictions for these partitions are given by the mean values of the dependent variables in that partition. The splitting criteria are applied to maximize the between-groups sum of squares in a simple analysis of variance:

$Max:SS_{T}-\left(SS_{L}+SS_{R}\right)$ (3)

Where $SS_{T}=\sum\left(y_{i}-\overline{y}\right)^{2}$ is the sum of squares for the tree node, and $SS_{R}$ and $SS_{L}$ are the sum of squares for the right and left son, respectively. A more effective approach to finding regression trees uses the ideal of cross-validation. The regression tree model was established in R 3.4.4 by the package of *rpart*.

S1.4 Model tree

A model tree is a technique for dealing with continuous class-learning problems, and was developed by Quinlan (Quinlan 1992). Compared with regression trees, the leaf of the mode l tree will contain a linear model based on some of the attribute values and yield a raw predicted value. There are two stages to building a model tree. In the first stage, the ideal decision tree is used to build a tree and minimize the variation for each subset partition. The splitting criterion is based on treating the standard deviation of the class value in the tree as a measure of the error at that node and calculating the expected reduction in error as a result of testing each attribute at that node. The attribute which maximizes the expected error reduction is chosen. The standard deviation reduction is calculated by

$SDR=sd\left(T\right)-\sum_{i} \frac{\left|T_{i}\right|}{\left|T\right|}\times sd\left(T_{i}\right)$ (4)

Where $T$ is the set of examples that reaches the node and $T_{1}$,$T_{2}$, …are the sets that result from splitting the node, according to the chosen attribute. In the present study, the M5 prime algorithm was used to establish a model tree, which was introduced in Wang and Witten (Wang and Witten 1996). We applied the R 3.4.4 to build the model tree.

S1.5 Combination forecasting model

The combination forecasting model aims to aggregate information from the different forecasting methods and combine them into a composite model. Furthermore, evidence from previous studies shows that there is more accuracy in combined forecasts, with little influence of the specific methods included in the combination (Bates and Granger 1969, Clemen 1989, Armstrong 2001, Aiolfi, Capistran et al. 2010). Generally, the combined forecast model is obtained by a linear combination of the sets of forecasts. Then, using a specific weight to combine these forecast methods, consideration is given to the relative accuracy of each mode and the covariances of forecast errors among the methods. Thus, the combination model is

$Y=\sum_{i=1}^{n} w_{i}f_{i}=w_{1}f_{1}+w_{i}f_{i}+...+w_{n}f_{n}$ (5)

Where $w_{i}$ is the weight of the *i*th forecasting model in the combination forecasting model, and the summarized weight, $\sum_{i=1}^{n} w_{i}=1,0\leq w_{i}\leq1,i=1,2...n$, $f_{i}$, is the prediction of *i*th forecasting model, with *Y* the prediction of the combined forecasting model. There are several methods used to calculate the weight of a single forecasting model, such as equal value method, standard deviation method and coefficient of variation method (Diebold and Lopez 1996, Timmermann 2006). It is acceptable that one of them would give the greater weight, which would seem to have lower errors. Therefore, we used the coefficient of the variation method to calculate the weights of the combined forecasting model, that is,

$V_{i}=\frac{\sigma_{i}}{\overline{x_{i}}}\left(i=1,2...n\right)$ (6)

Where $V_{i}$is the coefficient of variation of *i* th forecasting model, $\sigma_{i}$is the standard deviation of the *i*th forecasting model, and $\overline{x}_{i}$ is the mean value of the prediction. The weight for each forecasting model is calculated by

$W_{i}=V_{i}/\sum_{i=1}^{n} V_{i}$ (7)

In our study, four weights were calculated for four forecasting models, including the ridge regression *f_1_*, the partial least squares regression *f_2_*, the regression tree *f_3_* and the model tree *f_4_*. The form of combination forecasting model was given by

$Y=w_{1}f_{1}+w_{i}f_{2}+f_{3}+w_{n}f_{4}$ (8)

**Figure list**


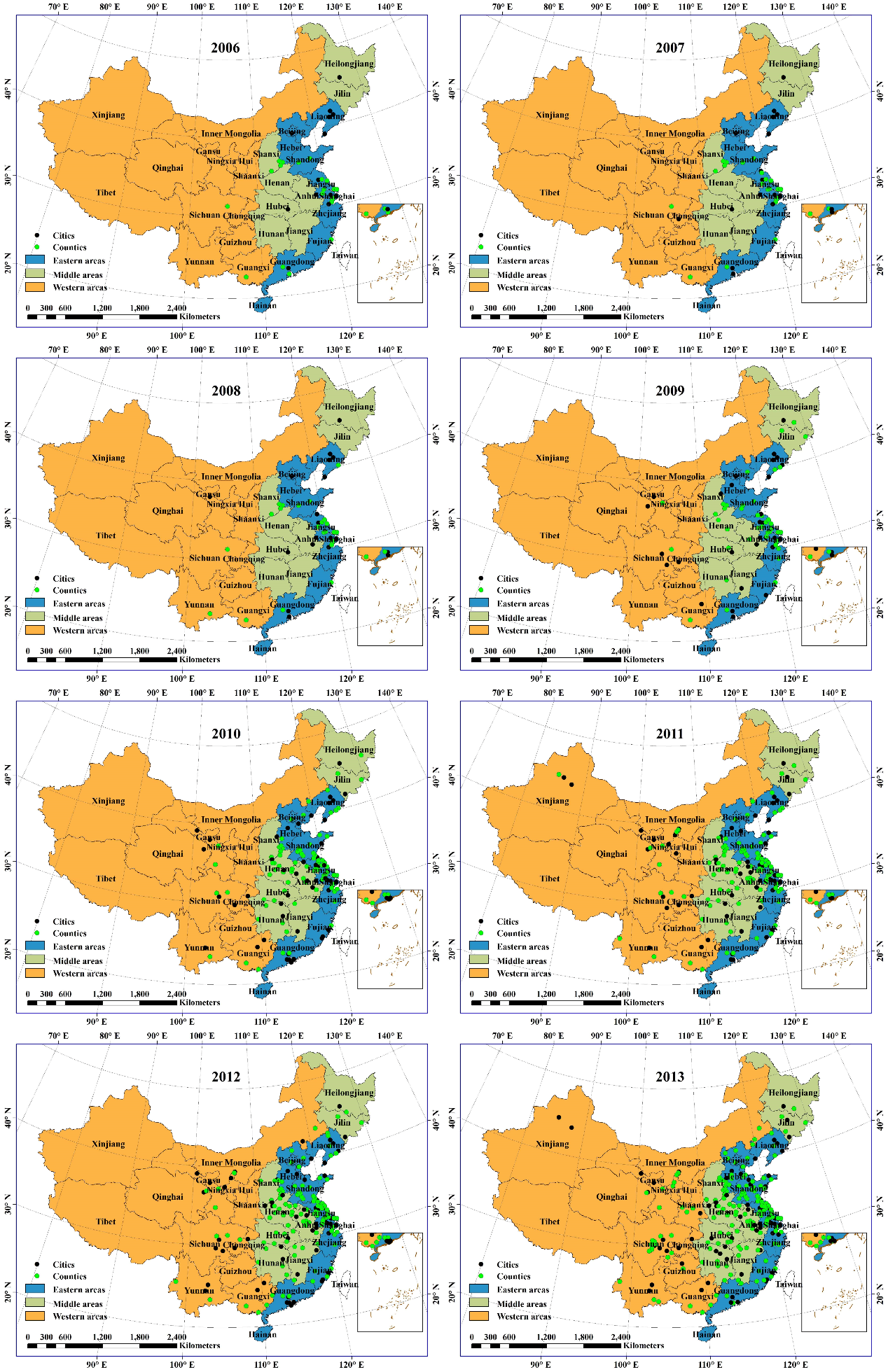


Figure S1. Spatial distribution of the cancer registries from 2006 to 2013 in China


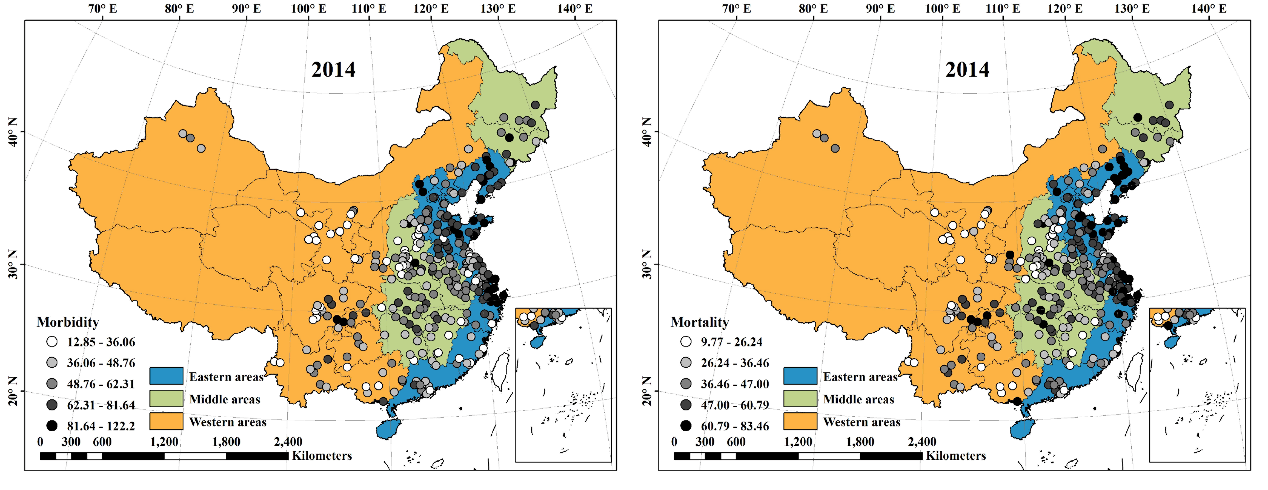


Figure S2. Spatial distribution of morbidity and mortality of lung cancer in 2014 across China


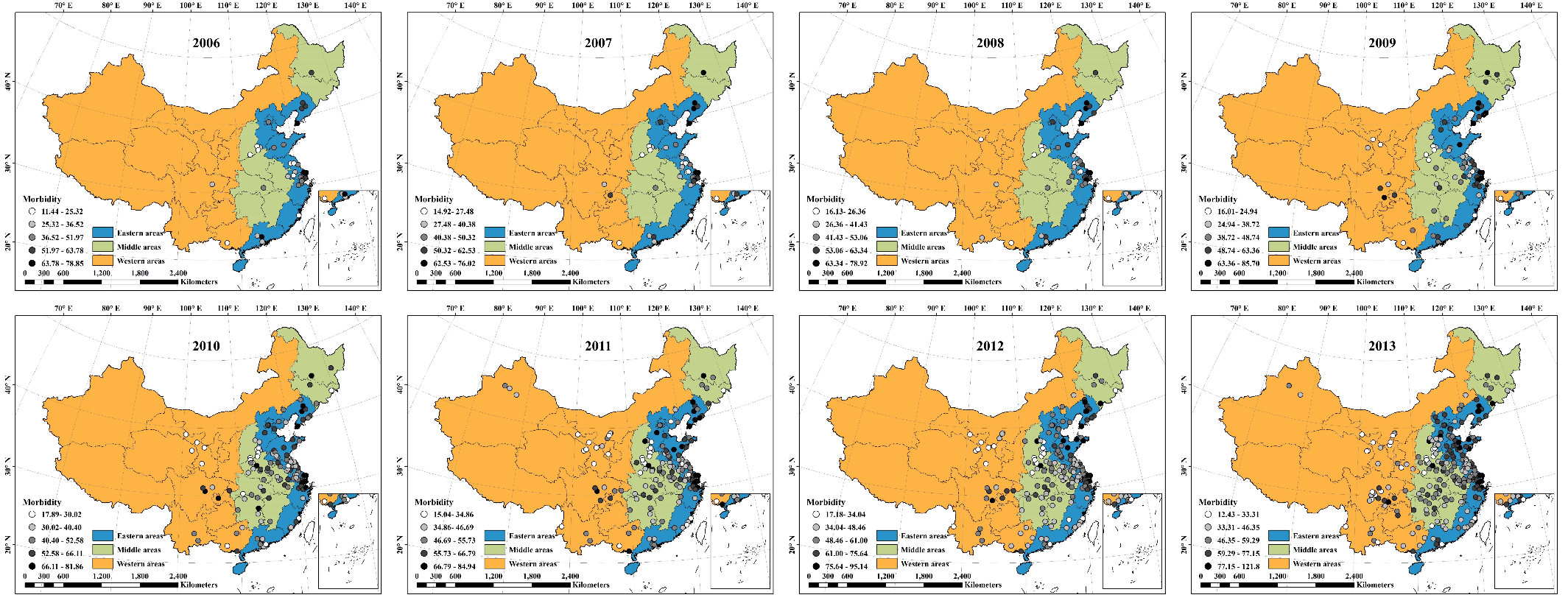


Figure S3. Spatial distribution of morbidity of lung cancer in China from 2006 to 2013.


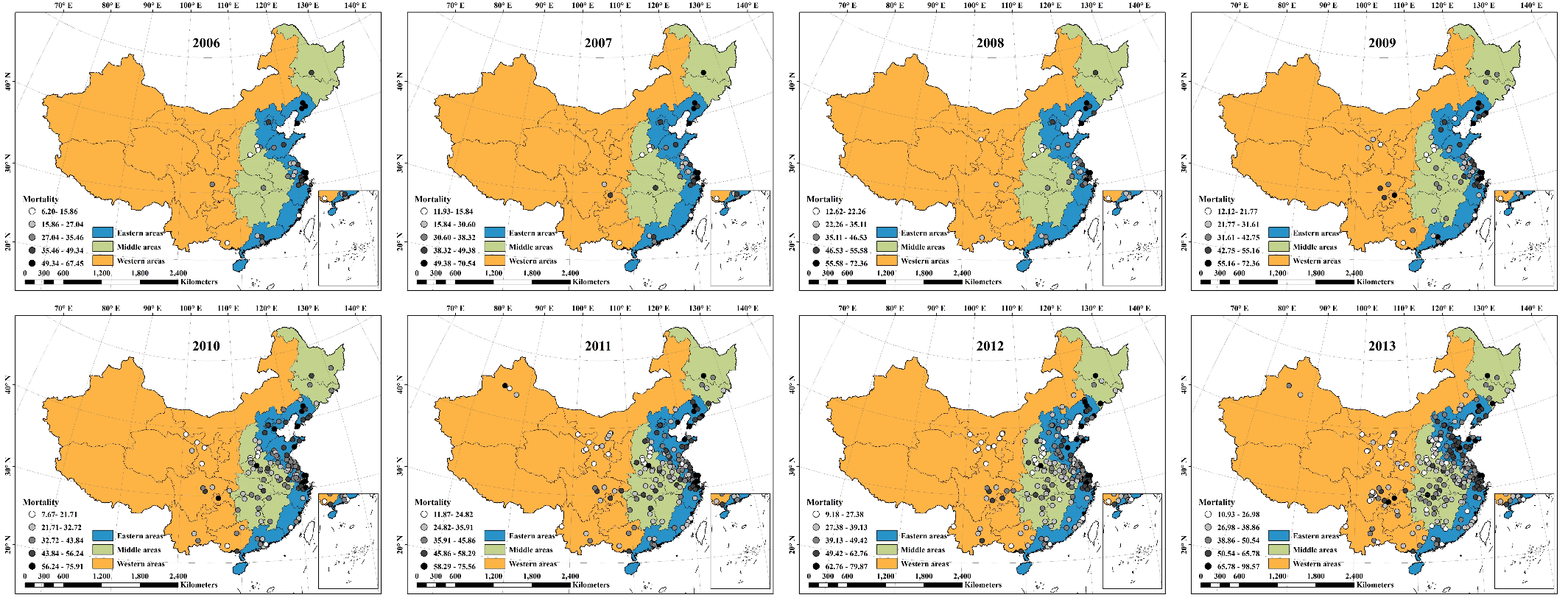


Figure S4. Spatial distribution of mortality of lung cancer in China from 2006 to 2013.

**Table list**

Table S1. The morbidity and mortality of lung cancer in urban and rural areas from 2006 to 2014

| Year | 2006 | 2007 | 2008 | 2009 | 2010 | 2011 | 2012 | 2013 | 2014 |
| --- | --- | --- | --- | --- | --- | --- | --- | --- | --- |
| Morbidity |  |  |  |  |  |  |  |  |  |
| All | 49.70 | 51.25 | 54.75 | 53.57 | 53.11 | 54.79 | 56.16 | 57.70 | 59.03 |
| Urban areas | 52.84 | 55.50 | 57.96 | 58.81 | 56.78 | 57.12 | 59.89 | 60.91 | 62.81 |
| Rural areas | 38.47 | 38.78 | 42.80 | 42.80 | 46.54 | 51.28 | 52.32 | 54.58 | 55.25 |
| Mortality |  |  |  |  |  |  |  |  |  |
| All | 44.15 | 45.50 | 46.07 | 45.57 | 44.03 | 45.48 | 45.76 | 46.92 | 47.23 |
| Urban areas | 47.29 | 49.56 | 48.76 | 50.32 | 47.44 | 48.37 | 49.77 | 50.47 | 50.28 |
| Rural areas | 32.93 | 33.58 | 36.03 | 35.81 | 37.92 | 41.13 | 41.63 | 43.47 | 44.17 |

Table S2. The Moran’s I statistics of lung cancer morbidity and mortality in China from 2006 to 2014.

|  | year | Moran's I | Z-Score | P-value |
| --- | --- | --- | --- | --- |
| Morbidity | 2006 | 0.26 | 3.58 | <0.0001 |
|  | 2007 | 0.32 | 3.22 | <0.001 |
|  | 2008 | 0.35 | 4.33 | <0.0001 |
|  | 2009 | 0.42 | 4.47 | <0.0001 |
|  | 2010 | 0.31 | 6.67 | <0.0001 |
|  | 2011 | 0.26 | 7.86 | <0.0001 |
|  | 2012 | 0.32 | 11.72 | <0.0001 |
|  | 2013 | 0.28 | 12.11 | <0.0001 |
|  | 2014 | 0.40 | 15.18 | <0.0001 |
| Mortality |  |  |  |  |
|  | 2006 | 0.41 | 5.34 | <0.0001 |
|  | 2007 | 0.42 | 4.11 | <0.0001 |
|  | 2008 | 0.42 | 5.16 | <0.0001 |
|  | 2009 | 0.44 | 4.64 | <0.0001 |
|  | 2010 | 0.36 | 7.82 | <0.0001 |
|  | 2011 | 0.27 | 8.12 | <0.0001 |
|  | 2012 | 0.36 | 13.09 | <0.0001 |
|  | 2013 | 0.25 | 11.03 | <0.0001 |
|  | 2014 | 0.44 | 16.98 | <0.0001 |

Table S3. The Moran’s I statistics of annual mean PM_2.5_ concentration in China from 2006 to 2014.

| year | Moran's I | Z-Score | P-value |
| --- | --- | --- | --- |
| 2006 | 0.27 | 3.74 | <0.0001 |
| 2007 | 0.21 | 2.23 | 0.026 |
| 2008 | 0.38 | 4.64 | <0.0001 |
| 2009 | 0.61 | 6.46 | <0.0001 |
| 2010 | 0.67 | 14.40 | <0.0001 |
| 2011 | 0.71 | 21.08 | <0.001 |
| 2012 | 0.62 | 22.09 | <0.0001 |
| 2013 | 0.53 | 22.73 | <0.001 |
| 2014 | 0.65 | 35.00 | <0.0001 |

Table S4. Model evaluation of five alternative forecasting models in four cross-validation experiments

| Mortality | 15% Cross-validation | | | | |  | 20% Cross-validation | | | | |
| --- | --- | --- | --- | --- | --- | --- | --- | --- | --- | --- | --- |
|  | RR | PL  SR | RT | MT | CFM |  | RR | PL  SR | RT | MT | CFM |
| MAE | 11.10 | 11.20 | 11.41 | 11.16 | 10.91 |  | 11.10 | 11.21 | 11.42 | 11.15 | 10.91 |
| MSE | 196.67 | 200.94 | 209.59 | 204.20 | 191.59 |  | 196.92 | 201.24 | 210.35 | 203.91 | 191.48 |
| MAPE | 0.27 | 0.28 | 0.29 | 0.30 | 0.27 |  | 0.27 | 0.28 | 0.29 | 0.30 | 0.27 |
| THEIL | 1.11 | 1.03 | 0.66 | 0.76 | 1.07 |  | 1.10 | 1.03 | 0.65 | 0.76 | 1.06 |
| BP | 0.01 | 0.01 | 0.01 | 0.01 | 0.01 |  | 0.01 | 0.01 | 0.01 | 0.01 | 0.01 |
| VP | 0.45 | 0.54 | 0.29 | 0.30 | 0.48 |  | 0.45 | 0.55 | 0.27 | 0.30 | 0.47 |
| CP | 0.45 | 0.45 | 0.71 | 0.69 | 0.71 |  | 0.45 | 0.45 | 0.72 | 0.69 | 0.72 |
| Morbidity |  |  |  |  |  |  |  |  |  |  |  |
| MAE | 12.79 | 12.81 | 13.12 | 13.13 | 12.52 |  | 12.80 | 12.83 | 13.19 | 13.15 | 12.54 |
| MSE | 268.96 | 269.79 | 284.89 | 289.53 | 261.29 |  | 269.52 | 271.02 | 288.39 | 290.67 | 262.30 |
| MAPE | 0.26 | 0.26 | 0.26 | 0.28 | 0.25 |  | 0.26 | 0.26 | 0.27 | 0.29 | 0.25 |
| THEIL | 1.12 | 1.01 | 0.70 | 0.68 | 1.07 |  | 1.12 | 1.01 | 0.67 | 0.68 | 1.05 |
| BP | 0.01 | 0.01 | 0.01 | 0.03 | 0.01 |  | 0.01 | 0.01 | 0.01 | 0.03 | 0.01 |
| VP | 0.45 | 0.50 | 0.28 | 0.28 | 0.47 |  | 0.45 | 0.50 | 0.26 | 0.27 | 0.47 |
| CP | 0.45 | 0.50 | 0.71 | 0.70 | 0.52 |  | 0.45 | 0.49 | 0.73 | 0.70 | 0.53 |

| 25% Cross-validation | | | | |  | 30% Cross-validation | | | | |
| --- | --- | --- | --- | --- | --- | --- | --- | --- | --- | --- |
| RR | PLSR | RT | MT | CFM |  | RR | PLSR | RT | MT | CFM |
| 11.10 | 11.22 | 11.46 | 11.17 | 10.91 |  | 11.10 | 11.22 | 11.50 | 11.19 | 10.91 |
| 197.12 | 201.77 | 212.41 | 204.96 | 191.72 |  | 196.80 | 201.67 | 214.70 | 205.53 | 191.93 |
| 0.28 | 0.28 | 0.29 | 0.30 | 0.27 |  | 0.27 | 0.28 | 0.29 | 0.30 | 0.27 |
| 1.10 | 1.03 | 0.63 | 0.75 | 1.05 |  | 1.11 | 1.03 | 0.60 | 0.74 | 1.03 |
| 0.00 | 0.00 | 0.00 | 0.01 | 0.01 |  | 0.00 | 0.00 | 0.00 | 0.01 | 0.01 |
| 0.46 | 0.55 | 0.25 | 0.29 | 0.47 |  | 0.45 | 0.56 | 0.23 | 0.30 | 0.46 |
| 0.45 | 0.44 | 0.74 | 0.69 | 0.74 |  | 0.45 | 0.44 | 0.76 | 0.69 | 0.76 |
|  |  |  |  |  |  |  |  |  |  |  |
| 12.80 | 12.86 | 13.26 | 13.15 | 12.56 |  | 12.80 | 12.87 | 13.33 | 13.15 | 12.57 |
| 270.12 | 273.06 | 291.85 | 291.10 | 263.40 |  | 269.68 | 274.04 | 294.70 | 290.83 | 263.39 |
| 0.26 | 0.26 | 0.27 | 0.30 | 0.25 |  | 0.26 | 0.26 | 0.27 | 0.28 | 0.26 |
| 1.12 | 1.00 | 0.64 | 0.68 | 1.04 |  | 1.13 | 0.99 | 0.62 | 0.68 | 1.03 |
| 0.00 | 0.00 | 0.00 | 0.03 | 0.01 |  | 0.00 | 0.00 | 0.00 | 0.03 | 0.01 |
| 0.45 | 0.51 | 0.24 | 0.28 | 0.46 |  | 0.45 | 0.51 | 0.23 | 0.28 | 0.46 |
| 0.45 | 0.49 | 0.75 | 0.70 | 0.53 |  | 0.45 | 0.48 | 0.77 | 0.70 | 0.54 |

Table S5. Standard deviation of model evaluation of five alternative forecasting models in four cross-validation experiments

| Mortality | 15% Cross-validation | | | | |  | 20% Cross-validation | | | | |
| --- | --- | --- | --- | --- | --- | --- | --- | --- | --- | --- | --- |
|  | RR | PLSR | RT | MT | CFM |  | RR | PLSR | RT | MT | CFM |
| MAE | 0.59 | 0.61 | 0.62 | 0.70 | 0.59 |  | 0.51 | 0.52 | 0.54 | 0.59 | 0.51 |
| MSE | 19.92 | 20.93 | 22.24 | 25.62 | 20.17 |  | 17.45 | 18.14 | 19.51 | 22.01 | 17.70 |
| MAPE | 0.02 | 0.02 | 0.02 | 0.07 | 0.02 |  | 0.01 | 0.01 | 0.01 | 0.07 | 0.01 |
| THEIL | 0.24 | 0.25 | 0.17 | 0.19 | 0.22 |  | 0.22 | 0.22 | 0.15 | 0.17 | 0.20 |
| BP | 0.01 | 0.01 | 0.01 | 0.02 | 0.01 |  | 0.01 | 0.01 | 0.01 | 0.02 | 0.01 |
| VP | 0.05 | 0.06 | 0.06 | 0.09 | 0.06 |  | 0.05 | 0.06 | 0.06 | 0.09 | 0.06 |
| CP | 0.05 | 0.06 | 0.06 | 0.09 | 0.06 |  | 0.05 | 0.06 | 0.06 | 0.09 | 0.06 |
| Morbidity |  |  |  |  |  |  |  |  |  |  |  |
| MAE | 0.68 | 0.69 | 0.72 | 0.83 | 0.69 |  | 0.58 | 0.60 | 0.64 | 0.79 | 0.61 |
| MSE | 29.65 | 30.19 | 33.38 | 38.03 | 30.42 |  | 25.70 | 26.64 | 29.39 | 35.26 | 26.76 |
| MAPE | 0.01 | 0.01 | 0.02 | 0.04 | 0.02 |  | 0.01 | 0.01 | 0.01 | 0.30 | 0.01 |
| THEIL | 0.25 | 0.22 | 0.16 | 0.18 | 0.21 |  | 0.23 | 0.20 | 0.14 | 0.16 | 0.19 |
| BP | 0.01 | 0.01 | 0.01 | 0.03 | 0.01 |  | 0.01 | 0.01 | 0.01 | 0.03 | 0.01 |
| VP | 0.59 | 0.61 | 0.62 | 0.70 | 0.59 |  | 0.51 | 0.52 | 0.54 | 0.59 | 0.51 |
| CP | 19.92 | 20.93 | 22.24 | 25.62 | 20.17 |  | 17.45 | 18.14 | 19.51 | 22.01 | 17.70 |

| 25% Cross-validation | | | | |  | 30% Cross-validation | | | | |
| --- | --- | --- | --- | --- | --- | --- | --- | --- | --- | --- |
| RR | PLSR | RT | MT | CFM |  | RR | PLSR | RT | MT | CFM |
| 0.44 | 0.45 | 0.47 | 0.53 | 0.45 |  | 0.39 | 0.40 | 0.43 | 0.50 | 0.39 |
| 15.21 | 15.75 | 17.05 | 20.24 | 15.47 |  | 13.63 | 14.19 | 15.73 | 19.28 | 13.66 |
| 0.01 | 0.01 | 0.01 | 0.05 | 0.01 |  | 0.01 | 0.01 | 0.01 | 0.05 | 0.01 |
| 0.21 | 0.21 | 0.13 | 0.17 | 0.18 |  | 0.20 | 0.20 | 0.12 | 0.16 | 0.16 |
| 0.01 | 0.01 | 0.01 | 0.02 | 0.01 |  | 0.01 | 0.01 | 0.01 | 0.02 | 0.01 |
| 0.05 | 0.05 | 0.06 | 0.09 | 0.06 |  | 0.05 | 0.05 | 0.05 | 0.09 | 0.05 |
| 0.05 | 0.05 | 0.06 | 0.09 | 0.06 |  | 0.05 | 0.05 | 0.05 | 0.09 | 0.05 |
|  |  |  |  |  |  |  |  |  |  |  |
| 0.51 | 0.53 | 0.55 | 0.70 | 0.52 |  | 0.46 | 0.48 | 0.50 | 0.65 | 0.47 |
| 22.80 | 24.28 | 26.18 | 32.09 | 23.59 |  | 20.42 | 21.90 | 23.25 | 29.20 | 20.98 |
| 0.01 | 0.01 | 0.01 | 0.36 | 0.01 |  | 0.01 | 0.01 | 0.01 | 0.05 | 0.01 |
| 0.21 | 0.19 | 0.13 | 0.16 | 0.18 |  | 0.21 | 0.18 | 0.12 | 0.15 | 0.17 |
| 0.01 | 0.01 | 0.01 | 0.03 | 0.01 |  | 0.01 | 0.01 | 0.01 | 0.03 | 0.01 |
| 0.05 | 0.06 | 0.06 | 0.07 | 0.05 |  | 0.05 | 0.07 | 0.05 | 0.08 | 0.05 |
| 0.05 | 0.06 | 0.06 | 0.08 | 0.05 |  | 0.05 | 0.07 | 0.05 | 0.08 | 0.05 |

**References**

Aiolfi, M., C. Capistran and A. Timmermann (2010). "Forecast combinations." CREATES research paper(2010-21).

Armstrong, J. S. (2001). Combining forecasts. Principles of forecasting, Springer**:** 417-439.

Bates, J. M. and C. W. J. Granger (1969). "COMBINATION OF FORECASTS." Operational Research Quarterly **20**(4): 451-&.

Breiman, L. (2017). Classification and regression trees, Routledge.

Clemen, R. T. (1989). "COMBINING FORECASTS - A REVIEW AND ANNOTATED-BIBLIOGRAPHY." International Journal of Forecasting **5**(4): 559-583.

Cule, E. and M. De Iorio (2012). A semi-automatic method to guide the choice of ridge parameter in ridge regression.

Diebold, F. X. and J. A. Lopez (1996). "8 Forecast evaluation and combination." Handbook of statistics **14**: 241-268.

Hoerl, A. E. and R. W. Kennard (1970). "RIDGE REGRESSION - BIASED ESTIMATION FOR NONORTHOGONAL PROBLEMS." Technometrics **12**(1): 55-&.

Hoerl, A. E., R. W. Kennard and K. F. Baldwin (1975). "RIDGE REGRESSION - SOME SIMULATIONS." Communications in Statistics **4**(2): 105-123.

Lawless, J. F. and P. Wang (1976). "SIMULATION STUDY OF RIDGE AND OTHER REGRESSION ESTIMATORS." Communications in Statistics Part a-Theory and Methods **A 5**(4): 307-323.

Quinlan, J. R. (1992). Learning with continuous classes.

Timmermann, A. (2006). "Forecast combinations." Handbook of economic forecasting **1**: 135-196.

Wang, Y. and I. H. Witten (1996). "Induction of model trees for predicting continuous classes."
